# Identification of *MSRA *gene on chromosome 8p as a candidate metastasis suppressor for human hepatitis B virus-positive hepatocellular carcinoma

**DOI:** 10.1186/1471-2407-7-172

**Published:** 2007-09-04

**Authors:** Ke-Feng Lei, Yan-Fang Wang, Xiao-Qun Zhu, Peng-Cheng Lu, Bing-Sheng Sun, Hu-Liang Jia, Ning Ren, Qing-Hai Ye, Hui-Chuan Sun, Lu Wang, Zhao-You Tang, Lun-Xiu Qin

**Affiliations:** 1 Liver Cancer Institute and Zhongshan Hospital, Institutes of Biomedical Science, Fudan University, Shanghai, China; 2 Statistics Graduate Program, Iowa State University, Ames, Iowa, USA

## Abstract

**Background:**

The prognosis of patients with hepatocellular carcinoma (HCC) still remains very dismal, which is mainly due to metastasis. In our previous studies, we found that chromosome 8p deletions might contribute to metastasis of HCC. In this study, we aimed to identify the candidate metastatic suppressor gene on chromosome 8p.

**Methods:**

Oligo-nucleotide microarrays which included 322 genes on human chromosome 8p were constructed to analyze the difference in gene expression profiles between HCC tissues with and without metastasis. The leading differentially expressed genes were identified and selected for further analysis by real-time PCR and Western blotting. Recombinant expression plasmid vectors for each target gene were constructed and transfected into HCC cells and its *in vitro *effects on proliferation and invasion of HCC cells were also investigated.

**Results:**

Sixteen leading differentially expressed genes were identified from the HCC tissues with metastasis compared with those without metastasis (*p *< 0.01, *q *< 16 %). Among of the 10 significantly down-regulated genes in HCC with metastasis, methionine sulfoxide reductase A (*MSRA*) had the lowest *p *value and false discovery rate (FDR), and was considered as a potential candidate for metastasis suppressor gene. Real-time PCR and Western blotting confirmed that the mRNA and protein expression levels of *MSRA *were significantly decreased in HCC with metastasis compared with those without metastasis (*p *< 0.001), and *MSRA *mRNA level in HCCLM6 cells (with high metastatic potential) was also much lower than that of other HCC cell lines. Transfection of a recombinant expression plasmid vector and overexpression of *MSRA *gene could obviously inhibit cell colony formation (4.33 ± 2.92 vs. 9.17 ± 3.38, *p *= 0.008) and invasion (7.40 ± 1.67 vs. 17.20 ± 2.59, *p*= 0.0001) of HCCLM6 cell line.

**Conclusion:**

*MSRA *gene on chromosome 8p might possess metastasis suppressor activity in HCC.

## Background

Primary liver cancer (mainly hepatocellular carcinoma, HCC) is one of the most frequent human cancers worldwide. The number of deaths from liver cancer is very similar to that of new cancer cases (598,000 and 626,000 respectively) and the liver cancer mortality rate is the third highest in the world [1]. In China, liver cancer kills 54.7 people out of one-hundred-thousand per year [2]. Although many advances have been made as a result of HCC clinical studies during the past decades that include early detection, surgical resection and liver transplantation, the general prognosis of the patients with HCC still remains dismal [3]. This outcome has been attributed to the high possibility of intra-hepatic metastases and recurrence after curative treatment [3-6].

Cancer metastasis is a highly complex multistep process that involves alterations in growth, dissemination, invasion and survival, which leads to subsequent attachment, angiogenesis, and growth of new cancer cell colonies [7]. Recently, the traditional metastasis paradigm has been challenged by the observations that most of the genetic and epigenetic changes necessary for metastasis appear to be the hallmarks of cancer [8-10].

In our previous studies, we found that chromosome 8p deletions might contribute to HCC metastasis [11]. This result was further confirmed by comparisons in nude mice models bearing human HCC with different metastatic potentials [12]. These findings provide new locus for exploring a new candidate metastasis suppressor of HCC. In our recent study on two HCC cell lines with different metastatic potentials, a novel metastatic suppressor gene, *HTPAP*, was identified on chromosome 8p12 [13]. On the basis of these findings, we aimed our current study to compare the difference in gene expression profiles between HCC tissues with and without metastasis, and to identify the candidate metastatic suppressor gene on chromosome 8p.

## Methods

### Human HCC specimens and cell lines

Human HCC tissues were obtained with informed consent from 60 patients who underwent liver resection for HCC at the Liver Cancer Institute and Zhongshan Hospital of Fudan University (Shanghai, China). These included 30 patients with metastatic HCC whose primary HCC lesions were accompanied by intrahepatic spreading, which had been regarded as the most frequently metastatic site of HCC [14], in portal vein (n = 26), hepatic vein (n = 2), or bile duct (n = 2), and 30 patients who had only solitary HCC without metastases. All patients had a history of hepatitis B virus (HBV) infection with an average age of 48.8 years, and 54 (90 %) of them were male. The diagnosis and histopathological features were confirmed to be HCC by pathologists. The tumor sizes of primary HCCs ranged from 1 cm to 25 cm in diameter with a median diameter of 7.8 cm.

Non-neoplastic liver tissues as references for microarrays assays were obtained from 10 patients who also had the history of HBV infection and underwent liver resection for haemangioma of liver. All specimens were frozen in liquid nitrogen immediately after resection and were stored at -80°C until use.

HCC cell lines, MHCC97-L, MHCC97-H, HCCLM3 and HCCLM6 were established from the same parent human HCC cell line, MHCC97, in authors' institute. They have an identical genetic background and stepwise increasing metastatic potentials, i.e., MHCC97-L had the lowest and HCCLM6 had the highest ability of metastasis [15,16]. They were cultured in Dulbecco's modified Eagle's medium (DMEM) (Gibco BRL, NY, USA) supplemented with 10 % (v/v) fetal calf serum (Hyclone, Utah, USA) at 37°C in a humidified incubator containing 5 % CO_2_.

This study was approved by the Ethics Committee of Zhongshan Hospital, Fudan University (Shanghai, China) (No. 20050310), and was in compliance with the Helsinki Declaration.

### RNA preparation

Total RNAs were extracted from each tissue sample and cell line using TRIZOL reagent (Life Technologies, Grand Island, USA) and was further purified with an RNeasy mini kit (Qiagen, Valencia, CA, USA) according to the manufacturer's instructions. The quality of the total RNA samples was determined by electrophoresis through formaldehyde agarose gels, and the 18S and 28S RNA bands were visualized under ultraviolet light.

### Microarrays experimental design

A reference design was used to compare the HCC with and without metastases indirectly [17]. RNA from 10 non-neoplastic liver tissues was pooled to generate the common reference sample. Twenty cases of HCCs with metastasis and 20 HCCs without metastasis were investigated against the common reference. Twenty metastatic HCCs used in microarray experiments were from patients with tumor spreading in portal vein (n = 18), hepatic vein (n = 1), or bile duct (n = 1) [see Additional file 1]. Dye swap was performed in each comparison. Therefore, considering the two floret types, two kinds of HCCs and biological replications, a total of 80 chips were used for comparing the gene expression pattern between the HCCs with and without metastasis.

### Description of 8p-specific microarrays

'8p-specific' microarrays were constructed using 322 genes on human chromosome 8p based on the UniGene clusters in GenBank [18]. In addition, 5 house-keeping genes and 8 intergenic sequences from the yeast were used as internal and external controls respectively. All of these 335 genes were spotted twice as duplicates on microarrays, and 6 blank spots were made as the negative controls. So, total 676 spots (26 × 26 matrix) were constructed on each '8p-specific' microarrays. Both the design of 70-mer oligonuclearotides for each gene and the construction of microarrays were made by CapitalBio Corporation (Beijing, China).

### Microarrays assays and statistic analysis

Five microgram of DNase-treated total RNA was used to prepare fluorescent dye-labeled cDNA through Eberwine's linear RNA amplification method followed with subsequent reverse transcription and Klenow enzyme reaction according to the previously described methods [19,20]. Procedures of hybridization and washing were described previously [20], arrays were scanned at 10-μm resolution by using a confocal LuxScan™ scanner (CapitalBio, Beijing, China), and the intensities of spots on each image were extracted with SpotData software (CapitalBio, Beijing, China). The LOWESS normalization method was employed to normalize the logarithm transformed background corrected signal intensities within each slide, thereafter, normalized data were median-centered for each channel. The 4 values for each gene for each specimen (twice spotted) on microarrays and dye swap were averaged as a signal intensity for each gene. Thus the difference of M (HCCs with metastases) and N (HCCs without metastases) was estimated by the ln M/ref-ln N/ref and T test were performed in SAS to compare the mean difference between metastatic HCC and non-metastatic HCC transcripts. In total, 322 fold change and p values were obtained and subsequently converted to a set of q-values using the method of Storey and Tibshirani [21]. The largest q-value in a list of genes declared to be differentially expressed provides an estimate of the False Discovery Rate (FDR), which is used as adjustment for multiple T test.

### RT-PCR

The synthesis of the first-strand cDNA was performed as described previously [22]. Target gene fragments were co-amplified with the internal control gene β-actin (991 bp) respectively. For the two co-amplified genes, amplification reaction was performed in the same tube comprising 10× PCR buffer, 25 mM MgCl_2_, 2 U Taq DNA polymerase and 10 pM of each of the flanking primers. The primer sequences were as follows: sense 5'- TGTACCAGCCAGAACACATG -3' and anti-sense 5'- CTGCTATCTTCACTCAGACC -3' for *MSRA*(621 bp); sense 5'- TGGGCATGGGTCAGAAGGA -3' and anti-sense 5'- AAGCATTTGCGGTGGACGATGGAGG -3' for *β-actin *(991 bp).

The PCR profile was 7 min at 95°C followed by 26 cycles of 30 s at 94°C, 30 s at 57°C, 45 s at 72°C, and a final extension of 10 min at 72°C. The RT-PCR products were determined by electrophoresis on 1.5 % agarose gel stained with ethidium bromide.

### Real-time PCR

All real-time PCR reactions were performed using a DNA Engine Opticon Thermal Cycler (MJ Research, Waltham, MA) and the SYBR Green PCR Core Reagents kit (PerkinElmer Applied Biosystems).

Regular RT-PCR was performed before real-time PCR to determine the amplification condition to have a specific amplification. Then real-time PCR was done in a final volume of 20 μl with 2 μl template cDNA (with a concentration of 20 ng/μl) with 10 μl Quantitect SybrGreen Kit (Qiagen, Valencia, CA) and 20 pM of each primer for target gene and housekeeping gene(*GAPDH*). The primer sequences were as follows: sense 5'-ATGCAGAAGTCGTCCGAGTG-3' and anti-sense 5'-TAGATGGCCGAGCGGTACTG-3' for *MSRA *(141 bp); sense 5'-ATGACCCCTTCATTGACC-3' and anti-sense 5'-GAAGATGGTGATGGGATTTC-3' for *GAPDH *(131 bp).

Activation was done with HotStar *Taq *DNA polymerase at 95°C for 15 min, followed by 45 cycles of 94°C for 15 s, 58°C for 30 s, and 72°C for 30 s then plate read. The Opticon Monitor Software package 1.02 was used for detection of fluorescent signals and Tm calculations. The transcripts of glyceraldehyde-3-phosphate dehydrogenase (*GAPDH*) gene, a reference gene for high abundance gene transcripts, were quantitated as the endogenous control to normalize each test sample.

Results of real-time PCR were represented as Ct values, where Ct was a fractional value defined as the cycle number at which the sample fluorescent signal passes a fixed threshold above the baseline. ΔCt was the difference in the Ct values derived from the specific genes being assayed and *GAPDH*, the N-fold differential expression in a specific gene of a tumor sample was expressed as 2^ΔCt ^[23].

### Western blotting

Protein was extracted by M-PER or T-PER (Pierce, USA), and protein concentrations were measured with the BCA protein assay kit (Pierce, USA). Total protein (50 μg) was separated by SDS-PAGE and transferred onto PVDF membranes. After being blocked in TBST with 50 ml/L skim milk for 2 h at room temperature, membranes were incubated with the primary antibody anti-MSRA (Upstate, USA) for 2 h. Anti-GAPDH (Santa Cruz, USA) was used as the endogenous control. The membranes were then washed thrice with TBST solution, followed by incubation for 1 h with HRP-linked secondary antibodies (Santa Cruz, USA) at room temperature. Finally, the membranes were visualized using the DAB reagent (Dako, Denmark).

### Construction and transfection of the recombinant plasmid of *MSRA *gene

The full-length cDNA sequence of *MSRA *gene was amplified by PCR, and inserted into the *Bam*HI and *Nhe*I sites of the expression plasmid vector pIRES2-EGFP. Its sequence was confirmed by DNA sequencing and the recombinant plasmids were named pIRES2-EGFP-MSRA. The recombinant plasmids were transfected into the HCCLM6 cell line using Lipofectamine 2000 according to the instructions from the manufacturer (Invitrogen, USA), with the empty plasmid transfection as control.

### Analysis of proliferation of HCC cells *in vitro*

Cell proliferation assays were performed by MTT (Promega, Madison, WI) colorimetry. Twenty-four hours after transfection, the HCC cells were treated with trypsin and harvested, then resuspended and plated in 96-well plates at 1 × 10^3 ^cells per well in 100 μL cell culture medium and maintained at 37°C in a humidified incubator containing 5% CO_2_. After every 24 h, 10 μL of the MTT solution was added into the triplicate wells and incubated at 37°C for another 4 h, 100 μL of DMSO was added and surged for 10 min to dissolve the crystal completely. Then absorbance at 540 nm was measured to calculate the numbers of vital cells in each well by ELISA reader.

The colony formation assay was performed to measure growth promotion by *MSRA *gene transfection into MHCC-LM6 cells according to our previously reported protocol [22]. Twenty-four hours after transient transfection by control (pIRES2-EGFP) and recombinant (pIRES2-EGFP- MSRA) plasmid vectors respectively, the transfected cells were treated with trypsin and harvested, washed, and resuspended in fresh complete medium. Identical numbers of empty or recombinant plasmid-transfected cells were plated in 6-well tissue culture plates to allow cells to form colonies. After 10 days incubation at 37°C in a humidified CO_2 _incubator, cells were fixed with 4% paraformaldehyde in PBS and stained with Giemsa stain. The cut-off point for colony size was ≥ 20 cells/colony. The number of colonies was counted within a field at 200× under a light microscope. For each test, a total of five fields were selected at random, and the numbers were averaged. The assay was repeated three times with 0.5 × 10^4^, 1 × 10^4^, and 2 × 10^4 ^of the cells seeded, respectively.

### *In vitro *Matrigel invasion assay of HCC cells

*In vitro *invasion assay were performed as our previously reported protocol [22]. Briefly, 100 μL of serum-free DMEM-diluted matrigel (0.8 mg/ml) (BD Biosciences, San Jose, Calif., USA) was added to the Transwell filters(8.0 μM pore size; Corning, N.Y., USA) of a Boyden chamber and incubated at 37°C for 2 h to form matrix gels. HCCLM6 cells (1 × 10^5^) transfected with recombinant plasmid vector (pIRES2-EGFP- MSRA) or pIRES2-EGFP vector were suspended in 100 μL serum-free DMEM and added to the top of the gels in the triplicate chambers. A mixture of 200 μL DMEM with 10% fetal calf serum, 200 μL supernatant of HCCLM6 cell culture, and 200 μL supernatant of NIH/3T3 cell culture was added to the lower chamber to serve as the chemoattractant. After 48 h of incubation at 37°C, the upper surface of the filters was carefully wiped with a cotton-tipped applicator, and the filters were fixed with 4% paraformaldehyde in PBS and stained with Giemsa stain. Cells invaded across the matrigel and passed through the Transwell filter pores toward the lower surface of the filters, and these were counted in five no overlapping 200× fields under a light microscope.

### Statistical analysis for real-time PCR and in vitro analysis

Statistical analyses for Real-time PCR and the in vitro analysis were performed with software from SPSS 10.0 for Windows (Chicago, IL., USA). Results of real-time PCR were evaluated by Mann-Whitney tests for two independent groups. The results of the cell proliferation assay, colony formation assay, and *in vitro *invasion assay were evaluated by Student's t-tests. A *p *value < 0.05 was taken as the level of significance.

## Results

### Identification of the metastasis-related genes for HCC

The '8p-specific' oligonucleotide microarrays, which were constructed with 322 genes and ESTs on human chromosome 8p, were used to investigate the different transcripts profiles between HCC with and with no metastasis. The data of array scanning were shown in Additional file 2. Statistical analyses, using two-group T-test based on the normalized expression levels, were performed to determine the differentially expressed genes. Of the 322 genes on the microarrays, 16 genes were found differentially expressed at a level of *p *< 0.01 between metastatic HCC and non-metastatic HCC, and the corresponding estimated false discovery rate (FDR) for *q *value thresholds was 16 % (Table 1). Among them, 10 genes were down-regulated, including Methionine Sulfoxide Reductase A (*MSRA*) as well as the other 3 well annotated genes (*WHSC1L1*, *ARHGEF10 *and *NAT1*). *MRSA *was significantly down-regulated (*p *= 0.00012, *q *= 3.5 %) in HCC with metastasis compared with those without metastasis. So, it was selected for the further confirmation and functional analysis.

**Table 1:** Differentially expressed genes between HCCs with metastases and without metastases detected by 8p-specific microarrays

Gene name	GenBank number	Fold change (HCC with metastasis/HCC without metastasis)	p	q
*MSRA*	NM_012331	0.55405	0.00012	0.03517
*LOC203076*	XM_114621.4	1.38670	0.00047	0.06824
*ATP6V1B2*	NM_001693	1.43027	0.00166	0.10781
*ARHGEF10*	NM_014629	0.54136	0.00181	0.10781
*LOC392188*	XM_373238	0.81971	0.00213	0.10781
*LOC389651*	XM_372039.1	0.65372	0.00259	0.10781
*UNQ9391*	NM_198464.1	1.26232	0.00287	0.10781
*NAT1*	NM_000662	0.71569	0.00296	0.10781
*LOC92755*	XM_047083.7	1.25717	0.00332	0.10781
*LOC389639*	XM_374258.1	0.74396	0.00371	0.10827
*LOC402328*	XM_378014.1	0.82349	0.00525	0.13363
*UNC5D*	NM_080872.1	1.90792	0.00549	0.13363
*TNKS*	NM_003747.1	1.12857	0.00709	0.15935
*LOC286097*	NM_181723.1	0.76227	0.00774	0.15970
*LOC389624*	XM_374248.1	0.68755	0.00874	0.15970
*WHSC1L1*	NM_023034	0.49360	0.00908	0.15970

### Down-regulated expression of *MSRA *in metastatic HCC samples and cell lines

**Figure 1:**
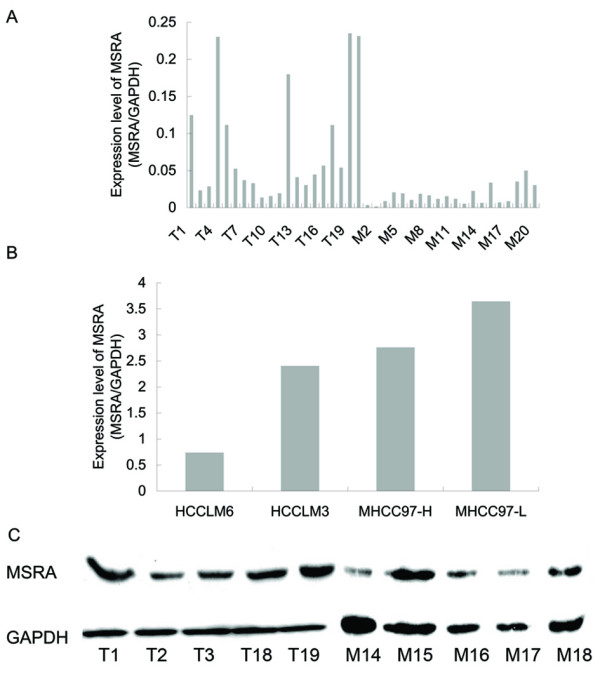
**Expression level of *MSRA *gene in the tumor tissues and cell lines of human hepatocellular carcinoma**. A and B: *MSRA *mRNA levels of clinical specimens (T1-20 were HCCs without metastases, M1-20 were HCCs with metastases) (A) and HCC cell lines (B) detected by real-time PCR. C: MSRA protein levels detected by Western blotting (T1 to T3, T18, and T19 were HCCs without metastasis, M14 to M18 were HCCs with metastasis).

In order to confirm the results of microarrays, the mRNA level of *MSRA *was examined in 40 HCC samples including 20 primary HCCs with metastasis and 20 HCCs without metastasis using real-time PCR. Twenty of them were from those used in microarrays assays and the others were newly selected. Since the expression level of *GAPDH *did not exhibit a significant difference between HCC with and without metastasis, it was used to normalize the *MSRA *expression data. Real-time PCR results confirmed that the expression level of *MSRA *in HCC with metastasis was obviously lower than that in HCC without metastasis (*p *< 0.001) (Figure 1A).

The mRNA levels of *MSRA *gene in MHCC97-L, MHCC97-H, HCCLM3 and HCCLM6 cell lines were also detected by real-time PCR. As shown in the Figure 1B, the expression level of *MSRA *in HCCLM6 cells (which had the highest metastatic ability) was significantly decreased compared with the other cell lines. Thus, the HCCLM6 cell line was selected for the further *in vitro *functional analysis of *MSRA *gene.

Western blotting was carried out with 5 HCCs without metastasis and 5 HCCs with metastasis to further confirm the protein expression level of *MSRA*. Consistent with the real-time PCR results, the protein expression level of MSRA in HCCs with metastasis was much lower than that in HCCs without metastasis (Figure 1C).

### *In vitro *effect of *MSRA *overexpression on proliferation and invasion of HCC cells

**Figure 2:**
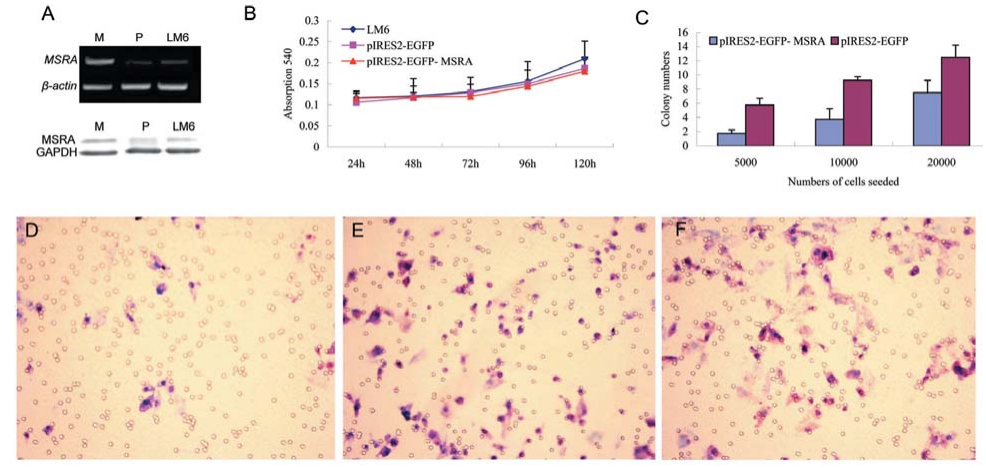
***In vitro *effect of *MSRA *overexpression on the proliferation and invasion of HCC cell line**. RT-PCR (A, upper) and Western blotting (A, low) showed that the expression level (both RNAs and proteins) was significantly higher in HCCLM6 transfected with pIRES2-EGFP-MSRA (*M*) compared with the control that transfected with pIRES2-EGFP (*P*) and HCCLM6 cell line (*LM6*). There was no significant difference in proliferation of HCC cells between the *MSRA*-transfected group and the controls (B), however, the colon formation in the recombinant vector-transfected cells were less than in the control (C). In *in vitro *matrigel invasion assay, the invasion ability of HCC cells was significantly decreased in HCCLM6 cells transfected with the *MSRA *gene (D), compared with those the transfected with empty vector (E) and the untransfected HCCLM6 cells (F).

To investigate the potential role of the *MSRA *gene on the invasiveness of HCC cells, the recombinant expression plasmid, named pIRES2-EGFP-MSRA, was constructed and transfected into the HCCLM6 cell line. Empty plasmids were also used as a control. There was no significant difference in the transfection efficiency between the recombinant and empty plasmids. RT-PCR and Western blot demonstrated that the expression levels of MSRA in the transfected cells increased dramatically as compared with the controls (Figure 2A).

The *in vitro *proliferation ability of HCCLM6 cells transfected with pIRES2-EGFP-MSRA was slightly inhibited, however, the difference did not reach statistical significance (0.13 ± 0.03 vs. 0.14 ± 0.03, *p *= 0.602) (Figure 2B). However, the ability of colony formation of HCCLM6 transfected with pIRES2-EGFP-MSRA was significantly decreased as compared with the controls (4.33 ± 2.92 vs. 9.17 ± 3.38, *p *= 0.008) (Figure 2C).

In the *in vitro *Matrigel invasion assays, the number of cells invaded through the transwell membrane in the pIRES2- EGFP- MSRA transfected group (7.40 ± 1.67) (Figure 2D) was significantly lower than those in the pIRES2-EGFP control group (17.20 ± 2.59, *p*= 0.0001) (Figure 2E). These suggested that *MSRA *might have the potential ability to inhibit cell invasion but have no significant effect on the proliferation of HCC cells.

## Discussion

Curative resection currently remains the major therapeutic method for HCC patients. However, due to the great possibility of tumor recurrence, caused mainly by metastasis, only definitive subsets of patients have the chance of being cured. Much like other kinds of solid malignant tumors, HCC metastasis is a complex interacting process between the host and cancer cells, and is regulated by multiple genes. Understanding the genes responsible for either enhancing or suppressing this process would allow novel diagnostic, therapeutic and prognostic applications in evolving and improving the clinical care of HCC patients. It has been demonstrated that more genetic aberrations exist during the process of cancer progression, invasion and metastasis [24,25]. These specific genomic aberrations could be used for mapping tumor suppressor genes [26], and provide clues for identifying metastasis-related genes. Many genes, such as *nm23*, *KAI1*, *KISS1*, *MKK4*, *BrMS1 *and so on, have been identified to have relations with the tumor metastasis [27,28].

In our previous studies, we found that HCC acquired a deletion on chromosome 8p as they progressed to metastatic stage, and that the chromosome 8p deletion may contribute to HCC metastasis [11]. This result was further confirmed by comparisons in nude mice models bearing human HCC with different metastatic potentials [12]. Using genome-wide microsatellite analysis, the deletion on chromosome 8p was further proven to be related to progression and metastasis of HCC, and that 8p23.3 and 8p11.2 were two regions harboring metastasis-related genes [29]. Similar results were obtained in other groups [30,31]. A deletion of chromosome 8p has also been shown to play an important role in the tumor progression and metastasis of many other kinds of human malignancies besides HCC including colorectal [32,33], bladder [34,35], breast [36], larynx [37], renal [38], and lung cancers [39]. Perhaps, 8p might harbor one or more tumor suppressor genes that are important in tumor progression and especially in the tumor metastasis of cancer including HCC [40].

Several candidate tumor suppressor genes have been identified from 8p including *DLC-1 *(8p21.3-22) [41], *FEZ1 *(8p22) [42], liver-related putative tumor suppressor (*LTPS*) gene (8p23) [43], *PCM1*(encoding a centrosomal protein) and *DUSP4*/*MKP-2 *(encoding a MAP kinase phosphatase) [44]. Alterations of these genes may occur as an early event in the development of cancer, and their association with metastasis is not confirmed [45] even though one very recent study indicated that *FEZ1 *may serve as a novel prognostic indicator for lung cancer [46]. It has been found that putative tumor suppressor genes that are mapped on chromosomes 8p21-22 may be involved in the metastasis of colorectal cancer. Allelic losses in these regions are possible risk factors for early lymph node metastasis [47]. Using a functional positional cloning strategy, Nihei et al. defined the region harboring the metastasis suppressor gene in 8p21-12, and localized it to a 60-kb cloned region [40].

To explore the new candidate metastatic suppressor of HCC, in our recent study on two HCC cell lines with different metastatic potentials, a novel metastatic suppressor gene, *HTPAP*, was identified on chromosome 8p12 [13]. However, regarding the limitation of study on cell lines and the development of human genome sequencing, in this study, we constructed '8p specific microarrays', which contain all of the genes and ESTs on human chromosome 8p, and used a number of clinical specimens to identify the genes relating to the metastasis of HCC. A total of 322 genes or ESTs were found from the UniGene clusters in GenBank and used for the construction of 8p-specific microarrays, a different number from the previous work, in which 100 ESTs were used [13]. Most of the functional genes have not been completely annotated yet. Sixteen genes were found differentially expressed between metastatic HCC and non-metastatic HCC. Among these sixteen genes, ten genes were down-regulated including four well- annotated genes, *MSRA, WHSC1L1, ARHGEF10 *and *NAT1*. Although *HTPAP *gene was significantly down-regulated in HCC cell line with high metastatic potentials (data not shown), and was also down-regulated in the metastatic HCCs, the difference did not reach the statistical criteria in this study. This suggested that there may be differences between cell lines and clinical samples, particularly for the high-throughputs assays.

*MSRA *was one of the four well- annotated genes that were significantly down-regulated in HCC with metastasis compared with those without metastasis; therefore, it was selected for the further confirmation and functional analysis.

The *MSRA *gene is located at chromosome 8p23.1. It has been found to express ubiquitously, with highest mRNA levels in adult kidney and cerebellum, followed by liver, heart ventricles, bone marrow and hippocampus. However, among the fetal tissues, the highest expression level was found in the liver [48]. In this study, both the mRNA and protein expression levels were decreased significantly in HCCs with metastasis. These results suggest that down-regulation of *MSRA *might play a role in the progression of HCC.

Furthermore, we investigated the effect of *MSRA *on the proliferation, colony formation and invasion ability of HCC cells in vitro. On the basis of high transfection efficiency, our studies showed that *MSRA *overexpression could slightly inhibit the proliferation ability of HCCLM6 cells, and more importantly, significantly inhibit the invasion ability. Although the *in vivo *experiments need to be done in order to further confirm the *MSRA *genes functions on HCC metastasis, these *in vitro *studies suggest that the *MSRA *gene might possess metastasis suppressor activity in HCC. Also, anti-tumor activity of MSRA was seen in a study about sulindac also[49].

Reactive oxygen can cause damage to many cellular components via DNA base alterations, strand breaks, damaging tumor suppressor genes. Secondly, oxidation also enhances the expression of proto-oncogenes, and plays a key role in the development of human cancer. MSRA has been implicated in protecting cells against oxidative damage, which can reduce methionine sulfoxide in proteins from oxidative damage to methionine [50]. MSRA has a broad specificity for oxidated compounds that contain a methyl sulfoxide group [51]. Oxidized methionine residues in different compartments are repaired by methionine sulfoxide reductases of different subcellular distribution regulated by alternative splicing [52].

The possible roles of MSRA in the development and progression of cancer are not yet fully understood. Hanbauer et al found that calcium phospholipid-binding protein (CPBP, synonyms: KLF6, ZF9, COPEB) can bind to the *MSRA *promoter at a 39-bp sequence of its 3' end. CPBP is a homologue of elongation factor-1 gamma, it can enhance *MSRA *gene expression [53], while wild-type p53 can enhance the activity of KLF6 [54]. Abnormalities of the p53 gene caused, by a wide range of cellular stresses including hypoxia are the most common molecular abnormality in human cancer, which were found in more than 50% of malignancies [55]. MSRA may be a point among the cyclic interconversion of MSRA, oxidation, p53 and CPBP. When oxidative damage occurs, p53, CPBP and MSRA are down-regulated in succession. Reactive oxygen can't be scavenged and promote the developing of malignant tumor. However, the real association of MSRA with oxidation, p53 and CPBP, the real mechanism of its effect on invasion and metastasis of HCC, are not clear yet, which deserves further study.

## Conclusion

In summary, *MSRA *gene, located on chromosome 8p23.1, was identified as a candidate of metastasis suppressor for HCC through chromosome 8p-specific microarrays analysis in HCC clinical samples. Both its mRNA and protein levels were down-regulated significantly in HCC with metastasis compared with HCC without metastasis. *In vitro *functional analyses suggested that *MSRA *could suppress the invasive ability of HCC cells. And further studies on the actual mechanisms of the effect on invasion and metastasis of HCC are suggested.

## Competing interests

The author(s) declare that they have no competing interests.

## Authors' contributions

KFL and YFW made the most important contributions to the manuscript, they performed most part of this study, including samples preparation, microarray assays and data analysis, and also writing the manuscript. PCL participated in microarrays data analyses. XQZ and BSS participated in *in vitro *functional analysis. HLJ, NR, QHY, HCS and LW participated in the collection of tissue samples and RNA preparation. ZYT was involved in overall design. LXQ conceived the study, participated in the study design, supervised the overall process and revised the manuscript. All authors have read and approved the final manuscript.

## Pre-publication history

The pre-publication history for this paper can be accessed here:



## Supplementary Material

Additional file 1 Clinical characteristics of patients with HCC enrolled in this study. The data represent the clinical characteristics of patients with hepatocellular carcinoma enrolled in this study, including patients' sex, age and serum AFP level, tumor location and size, Edmondson classification, vascular invasion, etc. Click here for file

Additional file 2 The raw data of microarrays. The data provided represent the signal intensities of each spot in the experiments of the microarrays of each examined sample. Click here for file
